# A Newly Authenticated Compound from Traditional Chinese Medicine Decoction Induces Melanogenesis in B16-F10 Cells by Increasing Tyrosinase Activity

**DOI:** 10.1155/2018/8485670

**Published:** 2018-11-18

**Authors:** Xiu Juan Xin, Jiahong Zou, Tao Zou, Huoli Shang, Li Yun Sun

**Affiliations:** State Key Laboratory of Bioreactor Engineering, East China University of Science and Technology, 130 Mei-Long Road, Shanghai 200237, China

## Abstract

Vitiligo is a kind of skin dysfunction on melanogenesis. The highly prevalent, chronic, and distinctive complexion changes on patients have imposed enormous psychic and economic burden on both individuals and society. Traditional Chinese Medicine (TCM) is a kind of precious source on chronic disease treatment, including skin dysfunctional diseases. In our previous study, a new compound named apigenin-7-butylene glucoside has been authenticated and purified from a prescription of Chinese traditional medicine formula which has been used clinically in vitiligo treatment. The aim of this work is to evaluate the effects of this compound on melanogenesis using melanoma cell B16-F10* in vitro*. The results showed that apigenin-7-butylene glucoside had almost no cytotoxicity on B16-F10 cells within a lower dose of 5.0 *μ*g ml^−1^ and enhanced the melanin level to about 41% and tyrosinase activity to 1.32-fold when compared with controls. The compound showed minor cytotoxicity to B16-F10 cells at the higher concentration of 10 *μ*g ml^−1^ and 50 *μ*g ml^−1^, the inhibition rate was 8.4% and 11.8%, and the melanin level and tyrosinase activity showed a decreased trend because of the lower cell number at the higher concentrations. The results indicated that apigenin-7-butylene glucoside was safe to B16-F10 cells within a lower concentration, <5.0 *μ*g ml^−1^. Incubated with 5.0 ug ml^−1^of apigenin-7-butylene glucoside for 48 hours, the mRNA and protein levels of* Tyr*,* Trp-1*, and* Trp-2* genes were all increased except* Mitf *in B16-F10 cells. The stimulation of apigenin-7-butylene glucoside on melanogenesis of B16-F10 cells through Tyr, Trp-1, and Trp-2 pathway highlighted the potential usage of the compound in vitiligo treatment.

## 1. Introduction

Vitiligo is a kind of depigmenting skin disease with an estimated prevalence of 1% among population worldwide [1]. The disease seems to affect all ethnic groups equally [2], and vitiligo has no race and gender predilection. Most patients with vitiligo characteristics are prior to the age of 20 years; it is stubborn and disfigure on the patients which can derive serious social problems [3]. The goal of vitiligo treatment is to suppress depigmentation and stimulate repigmentation of the patients. There have reports that the melanin level could be improved by suppressing the inflammation (e.g., topical corticosteroids and immunomodulators) or oxidation (e.g., vitamin D3 analogues and pseudocatalase) in early to active lesions and stimulate melanocyte differentiation or migration; the therapies included phototherapy like PUVA, narrowband UVB, 308 nm excimer laser, heliotherapy, and or vitamin D3 analogues [4]. Unfortunately, not all patients respond to the available therapies and some remedies have shown side effects which would suppress further treatment [5]. The Traditional Chinese Medicine (TCM) decoctions that have been widely used as externally applied agentin China showed efficacious and less side effects on vitiligo cure.

The TCM decoction used here composed by nine kinds of herbs which has been prevented for more than 60 years in China for its safety and reliability and even used on children or pregnant woman. After two courses of treatment with this prescription, the lesions skin areas could be recovered with melanocyte. The skin color disorders could be improved after further three courses of treatment and recurrence does not happen in the following years [6]. According to the Chinese medicine theory, the decoction can modulate the essential deficiency by promoting blood circulation and darken the skin color by balancing melanin cell proliferation and pigmentogenesis [7]. However, the possible respond compounds and the exact mechanisms of the melanogenesis induced by the decoction remained ambiguous. In our previous studies, a new compound authenticated as apigenin-7-butylene glucoside which was one member of flavonoids was extracted from the medicine formulas; the purpose of this study is to uncover the possible pharmacological activity of the compound using B16-F10 cells* in vitro *and to evaluate its potential value on vitiligo treatment.

As for the etiopathogenesis of vitiligo, numerous pathogenic factors have been reported, including autoimmunity suppress, neurogenic dysregulation, autocytotoxicity, and oxidative stress [8], and among those theories, the degeneration of melanin in melanocytes was thought to be the main cause of vitiligo formation [9].

Melanin biosynthesis was modulated by various cytokines, such as basic fibroblast growth factor, endothelin-1, cAMP response binding protein, and melanin stimulating hormone [10, 11]. Among these factors, tyrosinase (TYR) was the most restrictive factor in melanin biosynthesis. Tyrosinase is a rate-limiting enzyme, it catalyzes the oxidation of L-tyrosine to 3,4-dihydroxyphenylalanine (L-DOPA), and then L-DOPA is oxidated to produce dopaquinone and thus affects the production of melanin and the complexion of human beings. The tyrosinase was thought to be the key enzyme during melanogenesis [12, 13], so far, most of skin-whitening agents in cosmetics business are TYR inhibitors [14].

The following melanin biosynthetic processes after dopaquinone are catalyzed by tyrosinase-related protein 1 (TRP-1) and tyrosinase-related protein 2 (TRP-2) [15]. The expression level and activity of tyrosinase together with tyrosinase-related proteins in the melanocyte are also affecting the melanin formation process [16–18]. The protein levels of the TYP and TRPs are regulated by the microphthalmia-associated transcription factor (MITF) as reported [19–21]. MITF is an important transcription factor that regulates the expression levels of tyrosinase family, such as tyrosine, tyrosine-1, and tyrosine-2 and might indirectly participate in the proliferation of melanocytes, cell survival, and melanogenesis [22–24].

The purpose of this study is to explore the possible pharmacological activity of the newly authenticated compound from the useful decoction which has been used for vitiligo cure and to uncover the possible mechanisms of it on melanogenesis using B16-F10 cells* in vitro*. The decoction consists of nine crude herbs, including* Lithospermum Erythrorhizoni*,* Radix Salviae Miltiorrhizae*,* Cortex Lycii Radicis*,* Rhizoma Bletillae*,* Divaricate Saposhnikovia Root*,* Radix Angelicae Sinensis*,* Rhizoma Chuanxiong*,* Phryma Leptostachya,* and* Radix Glycyrrhizae*. After purification using affinity resin and cation exchange column of the ethyl acetate fraction, a new compound apigenin-7-butylene glucoside was extracted and authenticated by High Performance Liquid Chromatography (HPLC), mass spectrometry (MS), and nuclear magnetic resonance (^1^H-NMR). The results showed that it had faint cytotoxicity on B16-F10 cells but stimulated tyrosinase activity and melanin mass to about 1.4-fold of the controls. Moreover, it also promoted the transcription and expression levels of* Tyr*,* Trp-1*, and* Trp-2* genes in B16-F10 cells and does not affect the transcription and expression levels of the cytokine of* MITF* in B16-F10 cells. The results suggested that the apigenin-7-butylene glucoside stimulated melanogenesis activity of B16-F10 cells by activating the* Tyr*,* Trp-1*, and* Trp-2 *melanin biosynthesis pathway and showed potential values in vitiligo treatment.

## 2. Materials and Methods

### 2.1. Reagents and Cells Culture

RPMI 1640 with phenol red, fetal bovine serum (FBS), penicillin/streptomycin, and trypsin was purchased from Hyclone (South Logan, UT, USA). Dimethylsulfoxide (DMSO), thiazolyl blue tetrazolium bromide (MTT), Triton X-100, sodium hydroxide (NaOH), and L-3,4-dihydroxyphenylalanine (L-DOPA) were acquired from Shanghai Sangon Biotech (Shanghai, China). PrimeScript™ 1st strand cDNA Synthesis Kit and SYBR Premix Taq™ (Tli RNaseH) were purchased from TaKaRa (Dalian, China). *β*-actin antibodies and goat anti-rabbit peroxidase-conjugated secondary antibodies were purchased from Sigma–Aldrich (St. Louis, MO, USA). Antibodies against TRP-2, TRP-1, and MITF were purchased from Santa-Cruz Biotechnology (Santa Cruz, CA, USA). Tyrosinase antibody was purchased from Abcam (Cambridge, MA, USA). The other chemicals and reagents used in the study were high-grade commercial products.

The B16-F10 mouse melanoma cells line was purchased from the Cell Bank of Chinese Academy of Sciences (Shanghai, China). Cells were grown in RPMI 1640 media with phenol red, supplemented with 10% FBS, 100 Uml^−1^ penicillin, and 100 *μ*g ml^−1^ streptomycin in a dynamic incubation system at 37°C in a 5% CO_2_ (Thermo Fisher Scientific, USA), humidified atmosphere.

### 2.2. Cell Viability Assay

Cell viability analysis uses MTT assay. Briefly, B16-F10 cells inoculated into 96-well plates (5×10^3^ cells/100 *μ*L media/well) for 12 hrs. Different concentrations of stocked apigenin-7-butylene glucoside (0.625, 1.25, 2.5, 5.0, 10.0, and 50.0 ug ml^−1^) were added and incubated for another 48 hrs in the incubator. Then, 20*μ*l MTT (5 mg/ml in PBS) solution was added to each well and incubated at 37°C in a 5% CO_2_ humidified atmosphere for 4 hrs. MTT media were subsequently collected from all cells and the produced formazan crystals were solubilized with 150 *μ*L DMSO per well by shaking for 10 min at ~120 rpm. Then the absorbance at 570 nm was performed in a microplate spectrophotometer (Bio Rad, model 680). Absorbance of the cell samples without apigenin-7-butylene glucoside added set as negative control, and the cells survival was regarded as 100%. The viability of the treatment cells was expressed as (A_570_ treatment/A_570_ of control) × 100%. Each treatment group was performed in triplicate and each experiment was repeated for three times.

### 2.3. Tyrosinase Activity and Melanin Content Assay

Tyrosinase activity was determined by measuring the L-DOPA oxidation rate. Briefly, the cells were treated with different concentrations of apigenin-7-butylene glucoside for 48 hours, then washed twice with PBS (pH 7.2, 10 mM), and frozen at -80°C for 30 minutes in PBS (pH 7.2, 10 mM) containing 0.1% Triton X-100, after melting at room temperature. 100 *μ*L freshly prepared substrate solution (0.1% v/v L-3,4-dihydroxyphenylalanine) was added to each lysate and incubated for 2 hours at 37°C, and the levels of dopachrome were monitored under 475nm. Cells without the compound added were regarded as control, and the relative activity of tyrosinase was expressed as (A_475_ treatment/A_475_ of control) × 100%.

As for the melanin content analysis, 1N the NaOH solution (with 10% DMSO) was used to dissolve the melanin of the cells after treatment with different concentration of apigenin-7-butylene glucoside for 48 hours. The cells with alkaline were incubated at 80°C for 1 hour, the supernatant was collected, and the absorbance of melanin in the cell lysis was analyzed under 470 nm. The level of melanin was expressed as (A_470_ treatment/A_470_ of control) × 100%. Each treatment was performed in triplicate and each experiment was repeated for three times.

### 2.4. *Tyr*,* Trp*-1,* Trp*-2, and* Mitf* mRNA Level Determination

Total RNA was isolated using RNAiso kit. qRT-PCR analysis was performed for mRNA level analysis. The qRT-PCR protocol was conducted as follows: SYBR Premix Ex Taq™ (TakaRa Bio Inc Takara, Japan) kit used for double-stranded cDNA amplification. The cycling parameters were 1 cycle PCR reaction at 60°C for 2 minutes, then 1 cycle at 95°C for 10 min, and 30 cycles at 95°C for 30 s and 60°C for 30s. The C_t_ value of the target genes was measured during the exponential amplification phase. The relative expression levels (defined as fold change) of the target genes were determined using a 2^-△△Ct^ method, and *β-*actin was used as internal control. The expression level was normalized to the fold change corresponding to control cells, according to the levels of the housekeeping gene *β-actin*, defined as 100.0. In all cases, each sample was performed in triplicate and repeated for at least three times.

### 2.5. Western Blot Analysis

The cells were incubated with 1.25 ug ml^−1^, 2.5 ug ml^−1^, and 5.0 ug ml^−1^apigenin-7-butylene glucoside for 48 hours, then collected and washed with PBS ( pH7.2 10 mM) for twice, and then centrifuged 10 min at 3000rpm. Cells were lysed by RIPA kit (Beyotime Institute of Biotechnology, China). Proteins were quantified using BCA kit (Beyotime Institute of Biotechnology, China). Appropriate amounts of lysates (200 mg of total proteins per lane) were loaded onto SDS-polyacrylamide precast gels and ran at 120 V on ice. The samples were subsequently transferred from the gels onto polyvinylidene difluoride (PVDF) membranes. The membranes were subsequently washed with TTBS and blocked with 5%w/v skimmed milk for 1 hour at 25°C. The membranes were then incubated overnight at 4°C with the selected primary antibodies, including tyrosinase (1/1000), MITF (1/200), TRP-1 (1/200), TRP-2 (1/200), and *β*-actin antibodies. The membranes were then washed three times in Tween-Tris-buffered Saline (TTBS, 5 min per wash) and incubated with the secondary antibodies and goat anti-rabbit IgG conjugated with HRP at a dilution of 1:2000 for 1 hour at room temperature. Immunoblots were visualized using ECL Plus western blotting detection Kit (Amersham International, Little Chalfont, UK) and the signal density was calculated using a DNR Bio-Image system (Oclaro Israel Ltd., Jerusalem, Israel).

### 2.6. Statistical Analysis

The data were expressed as mean ± SEM. Multiple comparison statistical analysis was performed with one-way analysis of variance (ANOVA) followed by Tukey's post hoc test for correction.

## 3. Results

### 3.1. Cell Viability

**Figure 1:**
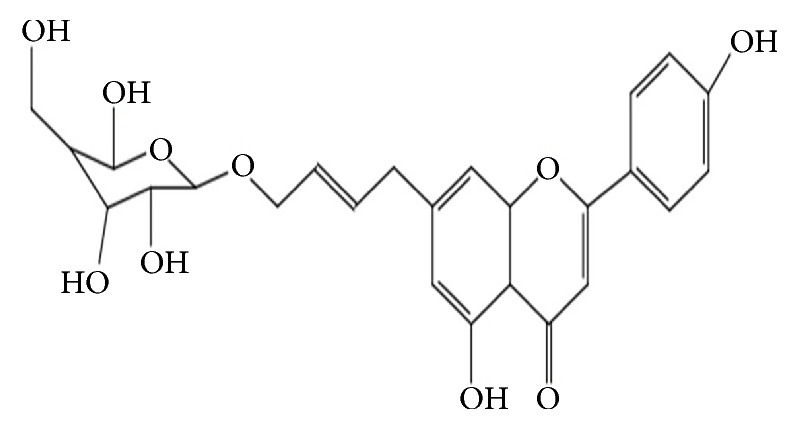
The chemical structure of apigenin-7-butylene glucoside.

**Figure 2:**
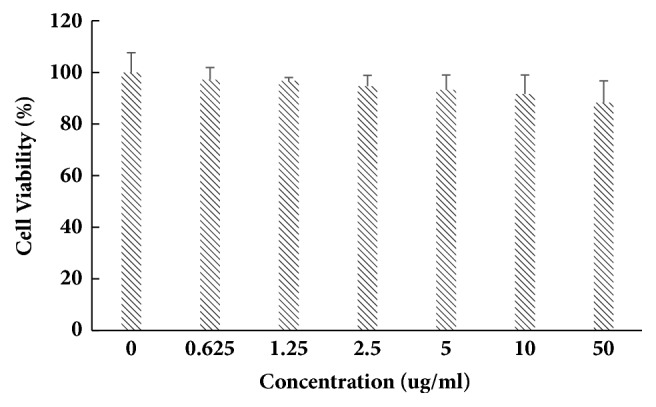
Cell availability test of B16-F10 after subjecting to different concentrations of apigenin-7-butylene glucoside (*∗* implies differences at p<0.005; *∗∗* implies significant differences at p<0.001).

The structure of apigenin-7-butylene glucoside was in Figure 1. The cell viability result showed that it had no cytotoxicity on B16-F10 cells within 50 *μ*gmL^−1^ after 48 hours of treatment. The viability of the B16-F10 cells was 97.1%, 96.7%,94.7%, and 93.3%, respectively, after being incubated with 0.625*μ*gmL^−1^, 1.25*μ*gmL^−1^, 2.5*μ*gmL^−1^, and 5.0*μ*gmL^−1^ for 48 hrs. Increasing the concentration to 10 and 50*μ*gmL^−1^, the viability rate of the cells was 91.6% and 88.2% (P>0.5) after 48 hours incubation, the inhibition rate was still lower than 15%, though the results showed a decreased trend with the increasing of compound concentration (Figure 2). The compound was safe to B16-F10 cells at a lower concentration and had minor cytotoxicity on B16-F10 cells even at higher concentration like 10 *μ*gmL^−1^ and 50*μ*gmL^−1^. The results suggested the safety of the compound at lower compound concentration (<5.0*μ*gmL^−1^) for B16-F10 cells.

### 3.2. Apigenin-7-Butylene Glucoside Stimulates Cellular Tyrosinase Activity and Melanin Levels

**Figure 3:**
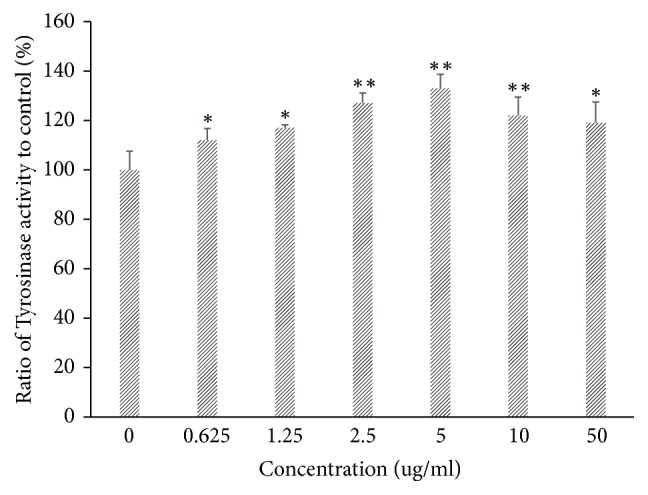
Tyrosinase activity evaluation of B16-F10 cells to different concentrations of apigenin-7-butylene glucoside (*∗* implies differences at p<0.005; *∗∗* implies significant differences at p<0.001).

**Figure 4:**
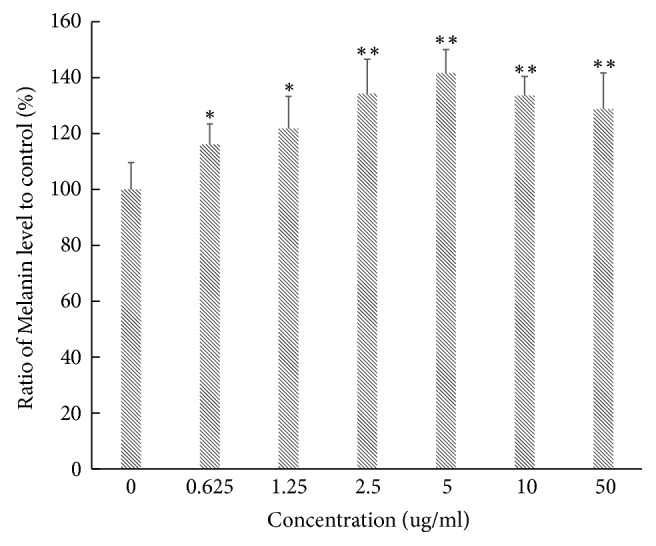
Melanin level analysis of B16-F10 cells to different concentrations of apigenin-7-butylene glucoside (*∗* implies differences at p<0.005; *∗∗* implies significant differences at p<0.001).

The tyrosinase activity and melanin level of the B16-F10 cells increased obviously after being incubating with the apigenin-7-butylene glucoside. The compound obviously enhanced tyrosinase activity in a dose dependent manner within a concentration of 5.0*μ*gmL^−1^. The tyrosinase activity increased to about 12% at a concentration of 0.625 *μ*gmL^−1^ compared with controls, and it increased to 17, 27, 32, and 22% at the concentrations of 1.25, 2.5, 5.0, and 10.0 *μ*gmL^−1^, respectively, when compared with the controls, as shown in Figure 3. Meanwhile, melanin levels of B16-F10 cells also promoted at all apigenin-7-butylene glucoside concentrations, the melanin level enhanced to 41.7% after incubated with 5.0*μ*gmL^−1^ apigenin-7-butylene glucoside compared with the control ones, as shown in Figure 4.

The tyrosinase activity and melanin level decreased after being incubated with higher compound concentration of 10 and 50 *μ*gmL^−1^ compared with 5.0 *μ*gmL^−1^, this might be the inhibition effect of the compound on B16-F10 cells, and there were decreased number of cells at the higher compound concentrations.

### 3.3. Apigenin-7-Butylene Glucoside Upregulates mRNA Level of Melanogenesis Pathway Genes

**Figure 5:**
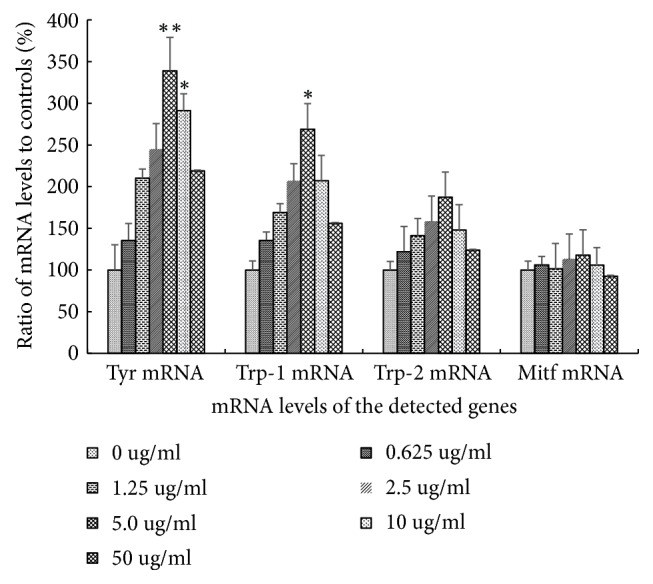
mRNA level assay of* Tyr*,* Trp*-1,* Trp*-2 and* Mitf* gene in B16-F10 cells after apigenin-7-butylene glucoside treatment (*∗* implies differences at p<0.005; *∗∗* implies significant differences at p<0.001).

The transcription levels of* Trp*,* MITF*,* Trp-1*, and* Trp-2 *genes were detected in this study which have been reported to participate in the melanin biosynthesis process. qRT-PCR assay results showed that the mRNA levels of* Trp*,* Trp-1*, and* Trp-2* genes were all increased, and the transcription levels of the detected genes showed a dose-depended manner within 5.0 *μ*gmL^−1^ as Figure 5 shown. Among the four genes,* Tyr* was much more sensitive to the stimulation of the apigenin-7-butylene glucoside on transcription level; it was 3.38-fold higher than that of controls after being incubated with 5.0 ugml^−1^apigenin-7-butylene glucoside for 48 hrs, and the* Trp*-1 also enhanced to about 2.69-fold at the same condition. As for* MITF* gene, no changes were detected after treatment with different concentration compound, as shown in Figure 5.

### 3.4. Protein Level Analysis of TYR, TRP-1, TRP-2, and MITF in B16-F10 Cells

**Figure 6:**
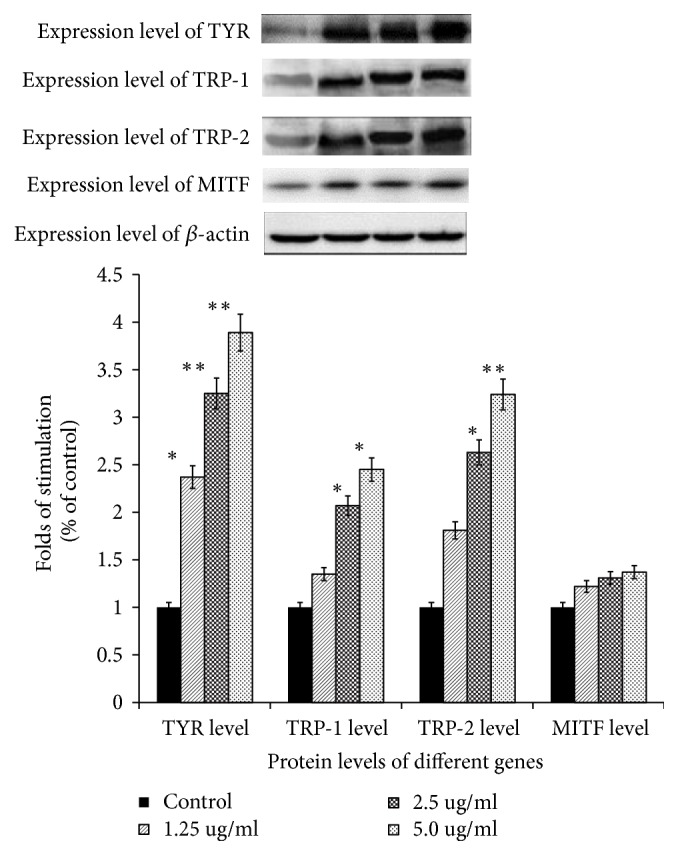
Expression level analysis of TYR, TRP-1, TRP-2 and MITF in B16-F10 cells after apigenin-7-butylene glucoside treatment (*∗* implies differences at p<0.005; *∗∗* implies significant differences at p<0.001).

The protein levels of TYR, TRP-1, TRP-2, and MITF were detected by western blotting analysis, the results showed that all the levels of detected protein increased compared with controls except MITF at a concentration of 5.0*μ*gmL^−1^, and the protein levels increased in a dose-depended manner for TYR, TRP-1, and TRP-2 within the lower compound concentrations as Figure 6 shown. Tyrosinase gene was more sensitive to the apigenin-7-butylene glucoside at the translational level than the others. The protein level of the tyrosinase after incubated with 5.0*μ*gmL^−1^apigenin-7-butylene glucoside for 48 hours was 3.89-fold higher than the controls, TRP-2 and TRP-1 level were also enhanced compared to the controls, and they were 3.24-fold and 2.45-fold. As for the MITF, the protein levels also increased slightly after incubation with the compound, but it was not assensitive as TYR, TRP-1, and TRP-2 do.

## 4. Discussion

Vitiligo is a kind of intractable skin disease, with dysfunction of melanin synthesis, and the lesions were distributed on mammalian skin. The abnormal physiological features can cause psychological stress and heavy economic burden on patients. Although several treatments are available, vitiligo cannot be completely cured for many patients [25], and some treatments can induce serious side affection on patients. Traditional Chinese Medicine (TCM) is one of the effective agents for vitiligo treatment in clinical and has been used to cure various human diseases for over 4000 years, including numerous skin dysfunctions [26]. Though most TCM prescriptions have attractive therapeutic effects, the application and popularization blocked for its magic composition and ambiguous pharmacological mechanisms.

The TCM formula studied in our work has been used in clinical for many years on vitiligo treatment in Henan province, China. The decoction of the formula has a high cure rate, but it is difficult to popularize out of the region for lack understanding of compound ingredients and unclear pharmacological mechanism. According to previous LC-MS spectrum results, the decoction of the formula herbs was composed mainly by flavonoid under the detection conditions, as other reported prescription on depigment treatment [27], and some responsible compounds were identified for pharmacology studies [28]. However, the responsible ingredients in the formula were not revealed still and thus attracted our interesting. Our group focused on identifying active compounds on melanogenesis of this formula previously and have fortunately isolated and authenticated a new compound of apigenin-7-butylene glucoside using HPLC and LC-MS spectrum techniques, after gradual purification with the decoction of the formula, 112 mg purified compound was extracted from 96 g herb formula (the detailed procedure reported in another paper).

In this work, we mainly evaluated the effects of apigenin-7-butylene glucoside on pigmentation induction and the underlying mechanisms that responded to vitiligo remedy using B16-F10 cells* in vitro*. The results indicated that apigenin-7-butylene glucoside could significantly upregulate melanin synthesis and had no obvious cytotoxicity on B16-F10 cells within 5.0 *μ*gmL^−1^ concentration. B16-F10 cells were incubated with 5.0*μ*gmL^−1^apigenin-7-butylene glucoside for 48 hours, 93.3% B16-F10 cells were available in Figure 3 and almost did not inhibit the growth of cells. Increasing the compound concentrations to 10 *μ*gmL^−1^ or 50 *μ*gmL^−1^, the proliferation rate was inhibited compared with the controls but still remained at a higher survive rate of 91.6 and 88.2%; there was no statistical difference on cells inhibition (P>0.5). The lower cytotoxicity of the drugs is significant as potential medicine agents [29]. Actually, it is common characters for many clinical compounds which showed contrast effects on detected targets at different concentrations. Arsenic trioxide (ATO) was a kind of poison; the epidemiological studies reported that drinking water or foods contaminated with low concentration of ATO might increase cancer risk [30] or fetal to people, but the high level ATO drinking water reduced markedly overall breast cancer mortality by over 50% d in the large patients during a 15-year contaminating period and in women under 60 by 70% [31]. The researcher found that, at therapeutic doses, ATO has an excellent safety profile for APL treatment even in children [32], although it has notorious toxicity at special doses due to its covalent binding to cellular targets [33].

In contrast to the lower cytotoxicity, the melanin levels increased obviously in B16-F10 cells, and it showed a dose-depended manner within the concentration of 5 *μ*gmL^−1^, and the melanin level increased to approximately 141.7% at a concentration of 5 *μ*gmL^−1^compared with the controls (untreated B16-F10 cells, P<0.005). Moreover, at higher concentrations of 10 *μ*gmL^−1^and 50 *μ*gmL^−1^, melanin level decreased slightly compared with 5*μ*gmL^−1^apigenin-7-butylene glucoside treatment, but they were still higher than controls, the melanin level was 133.6% and 128.7% (P<0.005), individually. The reason might be the less cell number when incubated with higher compound concentrations. Although the inhibition effects of the compound on B16-F10 cell were not notable, they were still established in our experiments.

Moreover, the transcription and expression levels of melanogenic enzymes TYR, TRP1, and TRP2 were all increased in B16-F10 cells after incubated with apigenin-7-butylene glucoside except the pigmentation associated transcription factor MITF.

The biosynthesis of melanin depended mainly on its precursor, tyrosine, in a common tyrosinase dependent way. The melanogenesis process was regulated by tyrosinase families, including tyrosinase (TYR), tyrosinase-related factors (TRP-1), and the cytokines* MITF* as reported [34]. Tyrosinase is the key enzyme in melanin biosynthesis and determines the color of skin and hair [35]. Tyrosinase modulates two key steps on melanin synthesis pathway, it catalyzes the hydroxylation of tyrosine to DOPA, and then DOPA is oxidized to dopaquinone; the two compounds are key products among melanin synthesis in the melanocytes and catalyzed by the same enzyme [34]. And so the tyrosinase active regulators have been clinically used for the treatment of several dermatologic disorders related to melanin extinction [36]. According to the melanogenesis theory, we speculated that the expression level and catalyst activity of tyrosinase families might have close relationship with melanin synthesis level. The present result showed that tyrosinase activity and melanin level in B16-F10 cells were enhanced after incubating within the detected conditions. The transcription and translation levels of* Tyr*,* Trp*-1, and* Trp*-2 genes in B16-F10 cells were also stimulated in this study (Figures 5 and 6). These results suggested that the apigenin-7-butylene glucoside stimulated melanogenesis in B16-F10 cells by enhancing TYR-TRP-1 and TRP-2 expression.

Microphthalmia-associated transcription factor (MITF), which is one of the most important nuclear activation transcription factors of tyrosinase, TRP-1 and TRP-2, plays key role in melanocyte development regulation and survival [37, 38]. Our results showed that apigenin-7-butylene glucoside had no effects on the MITF transcription and translation levels in B16-F10 cells as shown in Figures 5 and 6, though the MITF was reported to be the master regulator in the transcription of genes which were involved in melanin synthesis. Some reports believed that adiponectin and AICAR were well-known AMP-activated protein kinase (AMPK) activators; the compounds inhibited melanin synthesis through AMPK stimulating in melanocytes, but had no effects on MITF mRNA and translation levels [39].Actually, besides MITF modulated melanin synthesis pathway, many other signal transduction pathways were also disclosed on the balancing of melanogenesis process, like *α*-melanocyte-stimulating hormone (*α*-MSH) pathway and cAMP response element-binding protein (CREB) related pathway [40]. Alpha-MSH which is a kind of peptide hormone which bonds to melanocortin 1 receptor, when induced by other elements, will trigger the activity of adenylate cyclase via G proteins. The increased intracellular cAMP level then actives the phosphorylation of response element-binding protein at Ser133 via protein kinase A and enhances the expression of the transcription factors, like Lymphoid enhancer-binding factor 1 (LEF-1). The transcription factor LEF-1 then binds on the promoter of* Tyr* gene and enhances the gene expression. The dysregulation of LEF-1 will induce hyperpigmentary disorders [41]. Taken together, our findings suggested that the functional signaling moleculars on trigging the activity and expression levels of tyrosinase familiers needed further exploration.

There were many bioactive compounds derived from plants which have been used on regulating melanocytes growth and proliferation, and some agents have also been used for vitiligo treatment in clinical or for cosmetic purposes in commercially [42, 43]. This work focused on the bioactivities study of a newly authenticated compound of apigenin-7-butylene glucoside, and the results suggested its stimulating functions on melanogenesis in B16-F10 cells. Apigenin-7-butylene glucoside can stimulate the catalyse activity of tyrosinase and increase the transcription and expression level of* Tyr *familiar genes, including* Tyr*,* Trp-1*, and* Trp-2*. The enhancement effects of the compound on melanin synthesis were stimulated by the pathway of TYR-TRP, rather than the traditional MITF pathway. In conclusion, this study provided an interesting insight for potential mechanism studies of apigenin-7-butylene glucoside in vitiligo treatment in the future.

## Data Availability

The data used to support the findings of this study are included within the article.

## Abbreviations

TYR: Tyrosinase
TRP-1: Tyrosinase-related protein 1
TRP-2: Tyrosinase-related protein 2
MITF: Microphthalmia-associated transcription factor.
